# GrlR, a negative regulator in enteropathogenic *E. coli*, also represses the expression of LEE virulence genes independently of its interaction with its cognate partner GrlA

**DOI:** 10.3389/fmicb.2023.1063368

**Published:** 2023-02-16

**Authors:** Cristina Lara-Ochoa, Alejandro Huerta-Saquero, Abraham Medrano-López, Wanyin Deng, B. Brett Finlay, Ygnacio Martínez-Laguna, José L. Puente

**Affiliations:** ^1^Departamento de Microbiología Molecular, Instituto de Biotecnología, Universidad Nacional Autónoma de México, Cuernavaca, Mexico; ^2^Centro de Detección Biomolecular, Benemérita Universidad Autónoma de Puebla, Puebla, Mexico; ^3^Departamento de Bionanotecnología, Centro de Nanociencias y Nanotecnología, Universidad Nacional Autónoma de México, Ensenada, Mexico; ^4^Michael Smith Laboratories, Department of Microbiology and Immunology, and Biochemistry and Molecular Biology, University of British Columbia, Vancouver, BC, Canada; ^5^Vicerrectoría de Investigación y Estudios de Posgrado, Benemérita Universidad Autónoma de Puebla, Puebla, Mexico

**Keywords:** EPEC, type III secretion, LEE regulation, GrlR, GrlA, transcription, A/E pathogens, CAT reporter assay

## Abstract

**Introduction:**

Enteropathogenic *Escherichia coli* (EPEC), enterohemorrhagic *E. coli* (EHEC) and *Citrobacter rodentium* (CR) belong to a group of pathogens that share the ability to form “attaching and effacing” (A/E) lesions on the intestinal epithelia. A pathogenicity island known as the locus of enterocyte effacement (LEE) contains the genes required for A/E lesion formation. The specific regulation of LEE genes relies on three LEE-encoded regulators: Ler activates the expression of the LEE operons by antagonizing the silencing effect mediated by the global regulator H-NS, GrlA activates *ler* expression and GrlR represses the expression of the LEE by interacting with GrlA. However, despite the existing knowledge of LEE regulation, the interplay between GrlR and GrlA and their independent roles in gene regulation in A/E pathogens are still not fully understood.

**Methods:**

To further explore the role that GrlR and GrlA in the regulation of the LEE, we used different EPEC regulatory mutants and *cat* transcriptional fusions, and performed protein secretion and expression assays, western blotting and native polyacrylamide gel electrophoresis.

**Results and discussion:**

We showed that the transcriptional activity of LEE operons increased under LEE-repressing growth conditions in the absence of GrlR. Interestingly, GrlR overexpression exerted a strong repression effect over LEE genes in wild-type EPEC and, unexpectedly, even in the absence of H-NS, suggesting that GrlR plays an alternative repressor role. Moreover, GrlR repressed the expression of LEE promoters in a non-EPEC background. Experiments with single and double mutants showed that GrlR and H-NS negatively regulate the expression of LEE operons at two cooperative yet independent levels. In addition to the notion that GrlR acts as a repressor by inactivating GrlA through protein-protein interactions, here we showed that a DNA-binding defective GrlA mutant that still interacts with GrlR prevented GrlR-mediated repression, suggesting that GrlA has a dual role as a positive regulator by antagonizing GrlR’s alternative repressor role. In line with the importance of the GrlR-GrlA complex in modulating LEE gene expression, we showed that GrlR and GrlA are expressed and interact under both inducing and repressing conditions. Further studies will be required to determine whether the GrlR alternative repressor function depends on its interaction with DNA, RNA, or another protein. These findings provide insight into an alternative regulatory pathway that GrlR employs to function as a negative regulator of LEE genes.

## Introduction

Enteropathogenic *Escherichia coli* (EPEC) is one of the main etiological agents of severe diarrhea in children under 2 years of age, predominantly in developing countries (Pearson et al., 2016). EPEC, enterohemorrhagic *E. coli* (EHEC) and *Citrobacter rodentium* belong to a group of pathogens that possess the ability to induce a unique histopathological lesion known as attaching and effacing (A/E) (Croxen and Finlay, 2010). The localized destruction of the microvilli of intestinal epithelial cells followed by rearrangements of the cytoskeleton beneath the site of bacterial adherence, leading to the formation of actin-rich cup-like structures that favor an intimate interaction between the bacterium and the host cell, are hallmarks of this lesion (Spears et al., 2006; Frankel and Phillips, 2008).

Most genes required for A/E lesion formation are located within a pathogenicity island known as the locus of enterocyte effacement (LEE) (Gaytan et al., 2016). The LEE region contains five polycistronic operons (*LEE1*-*LEE5*), two bicistronic operons (*espG*-*rorf1* and *grlRA*) and four transcriptional units (*etgA*, *cesF*, *map*, and *escD*). *LEE1* to *LEE3* encode the structural components of a type III secretion system (T3SS) responsible for translocating effector proteins into the enterocyte. The *LEE4* operon codes for translocator proteins (EspA, B, D), and *LEE5* encodes proteins involved in the intimate attachment (intimin and Tir). Genes encoding effector proteins, chaperones and transcriptional regulators are distributed within and outside the LEE (Pearson et al., 2016; Serapio-Palacios and Finlay, 2020). The cooperative action of the EPEC translocated effector proteins leads to cytoskeleton rearrangements, increased cell permeability, decreased absorption of ions and nutrients, alterations of tight junctions and modulation of the inflammatory response in the host intestinal cells and thus diarrheal disease (Guttman and Finlay, 2008; Croxen et al., 2013).

**FIGURE 1:**
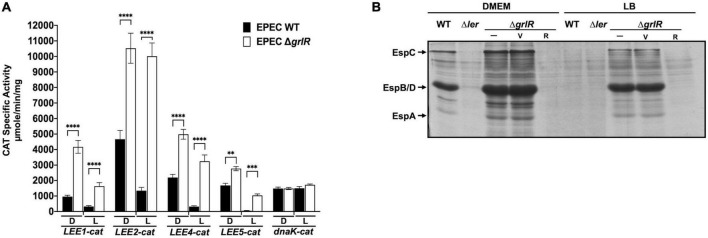
The absence of GrlR derepresses the expression of locus of enterocyte effacement (LEE) operons under repressing conditions. **(A)** The transcriptional activity of the *LEE1-cat*, *LEE2-cat*, *LEE4-cat*, *LEE5-cat*, and *dnaK-cat* fusions was analyzed in WT EPEC (black bars) and its Δ*grlR* isogenic mutant (white bars), grown in 50 ml DMEM (D) or LB medium (L) with shaking at 37°C. Specific chloramphenicol acetyltransferase (CAT) activity was determined from samples collected at an OD_600_ of 1. Values are an average of three independent experiments performed in duplicate. Error bars indicate standard deviations. Statistically different values are indicated (^**^p-value < 0.01; ^***^p-value < 0.001; ^****^p-value < 0.0001). **(B)** Profile of secreted proteins of EPEC WT, Δ*ler* and Δ*grlR* (carrying the empty vector pMPM-T3 or its derivative pT3GrlR) grown under the same conditions as in panel **(A)**. Secreted proteins were concentrated from culture supernatants by precipitation with trichloroacetic acid (TCA) and separated by 12% SDS-PAGE. V: pMPM-T3, R: pT3GrlR.

**FIGURE 2:**
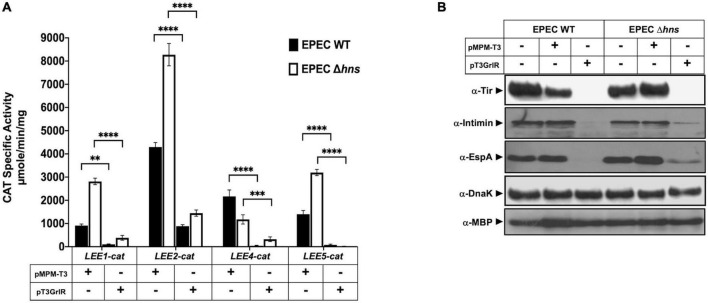
GrlR represses the expression of locus of enterocyte effacement (LEE) genes in the absence of H-NS. **(A)** Expression of the *LEE1-cat, LEE2-cat*, *LEE4-cat*, and *LEE5-cat* fusions was analyzed in WT enteropathogenic *Escherichia coli* (EPEC) (black bars) and its Δ*hns* isogenic mutant (white bars) carrying the empty vector pMPM-T3 (V) or its derivative pT3GrlR (R), grown in DMEM with shaking at 37°C. Specific chloramphenicol acetyltransferase (CAT) activity was determined using samples collected from cultures grown in 50 ml Dulbecco’s modified Eagle’s medium (DMEM) or Lysogeny Broth (LB) at an OD_600_ of 1. Values are an average of three independent experiments performed in duplicate. Error bars indicate standard deviations. Statistically different values are indicated (^**^p-value < 0.01; ^***^p-value < 0.001; ^****^p-value < 0.0001). **(B)** Total extracts were prepared from the same culture samples and separated by 12% SDS-PAGE. The expression of Tir, intimin and EspA was analyzed by western blotting using polyclonal anti-intimin and anti-EspA and monoclonal anti-Tir antibodies. As controls for protein loading, maltose binding protein (MBP) and DnaK were also detected using monoclonal anti-DnaK and polyclonal anti-MBP antibodies.

**FIGURE 3:**
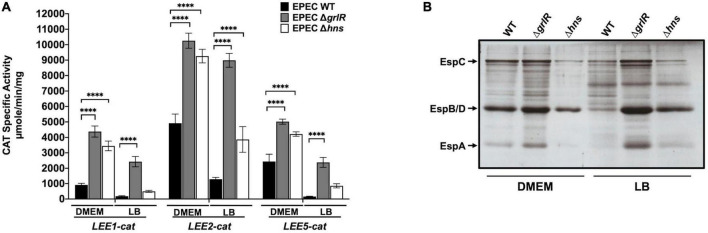
GrlR is the main repressor of locus of enterocyte effacement (LEE) gene expression under repressing conditions. **(A)** Expression of the *LEE1-cat*, *LEE2-cat* and *LEE5-cat* fusions was analyzed in WT EPEC (black bars) and its Δ*grlR* (gray bars) and Δ*hns* (white bars) isogenic mutants, grown in 50 ml of Dulbecco’s modified Eagle’s medium (DMEM) or Lysogeny Broth (LB) with shaking at 37°C. Specific chloramphenicol acetyltransferase (CAT) activity was determined from samples collected from cultures at an OD_600_ of 1. Values are an average of three independent experiments performed in duplicate. Error bars indicate standard deviations. Statistically different values are indicated (^****^p-value < 0.0001). **(B)** Secreted proteins of the same cultures were concentrated from supernatants by precipitation with trichloroacetic acid (TCA), separated by 12% SDS-PAGE and stained with Coomassie brilliant blue.

**FIGURE 4:**
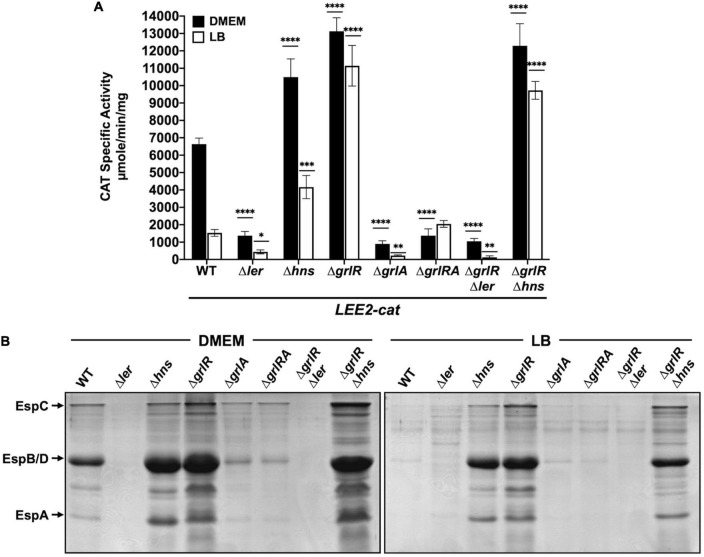
GrlR and H-NS repress locus of enterocyte effacement (LEE) gene expression by an indirect cooperative mechanism. **(A)** chloramphenicol acetyltransferase (CAT) activity of the *LEE2-cat* fusion was determined in WT enteropathogenic *Escherichia coli* (EPEC) and its Δ*ler*, Δ*hns*, Δ*grlR*, Δ*grlA*, Δ*grlRA*, Δ*grlR*Δ*ler*, and Δ*grlR*Δ*hns* derivative mutants grown in 50 ml of Dulbecco’s modified Eagle’s medium (DMEM) (black bars) or Lysogeny Broth (LB) (white bars) with shaking at 37°C. Specific CAT activity was determined from samples collected at an OD_600_ of 1. Values are an average of three independent experiments performed in duplicate. Error bars indicate standard deviations. Statistically different values are indicated (*p-value < 0.1; ^**^p-value < 0.01; ^***^p-value < 0.001; ^****^p-value < 0.0001). **(B)** Secreted proteins of the same cultures were concentrated from supernatants by precipitation with TCA, separated by 12% SDS-PAGE and stained with Coomassie brilliant blue.

**FIGURE 5:**
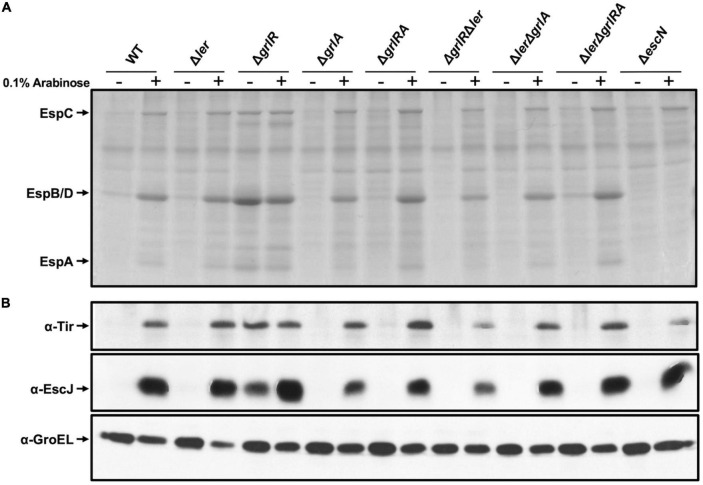
Protein secretion profile in the presence of a dominant negative H-NS mutant. **(A)** Secreted proteins from WT enteropathogenic *Escherichia coli* (EPEC) and its Δ*ler*, Δ*grlR*, Δ*hns*, Δ*grlA*, Δ*grlRA*, Δ*grlR*Δ*ler*, Δ*ler*Δ*grlA*, Δ*ler*Δ*grlRA*, and Δ*escN* derivative mutants, carrying plasmid pT6-HNS/G113D expressing H-NS^G113D^, grown in 50 ml Lysogeny Broth (LB) with (+) and without (–) 0.1% arabinose at 37°C with shaking, were concentrated from culture supernatants by precipitation with trichloroacetic acid (TCA), separated by 12% SDS-PAGE and stained with Coomassie brilliant blue. **(B)** Total extracts were prepared from the corresponding bacterial pellets and separated by 12% SDS-PAGE. Tir and EscJ expression was analyzed by western blotting using an anti-Tir monoclonal antibody and anti-EscJ polyclonal antibodies. As a control for protein loading, GroEL expression was also analyzed using an anti-GroEL monoclonal antibody.

**FIGURE 6:**
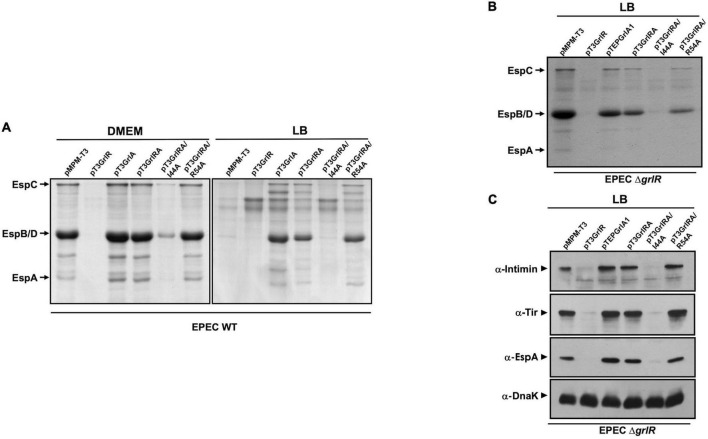
GrlA antagonizes GrlR-mediated repression. **(A)** Profile of secreted proteins from WT enteropathogenic *Escherichia coli* (EPEC) carrying the empty vector pMPM-T3 or its derivatives pT3GrlR, pTEPGrlA1, and pT3GrlRA, pT3GrlRA/I44A and pT3GrlRA/R54A grown in 50 ml Dulbecco’s modified Eagle’s medium (DMEM) or Lysogeny Broth (LB) to an OD_600_ of 1. Secreted proteins were concentrated from culture supernatants by precipitation with trichloroacetic acid (TCA), separated by 12% SDS-PAGE and stained with Coomassie brilliant blue. **(B)** Secreted proteins from EPEC Δ*grlR* carrying the same plasmids as in panel **(A)** grown in 50 ml of LB with shaking at 37°C. **(C)** Total extracts were prepared from bacterial samples of the same cultures described in panel **(B)**. Proteins were separated by 12% SDS-PAGE and expression of intimin, Tir and EspA was analyzed by western blotting using anti-intimin, anti-Tir and anti-EspA antibodies. As a control for protein loading, DnaK expression was also analyzed using anti-DnaK antibodies.

**FIGURE 7:**
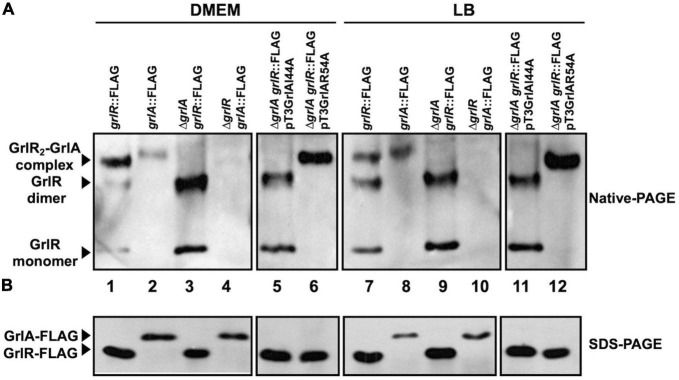
Analysis of GrlR_2_-GrlA complex formation by native gel electrophoresis. **(A)** GrlR_2_-GrlA protein complex formation in the enteropathogenic *Escherichia coli* (EPEC) strains *grlR*:3xFLAG, *grlA*:3xFLAG, Δ*grlR_grlA*:3xFLAG, *grlR*:3xFLAG_Δ*grlA*, as well as in the *grlR*:3xFLAG_Δ*grlA* strain carrying plasmids pTEPGrlA1/I44A or pTEPGrlA1/R54A, was analyzed from samples taken from cultures grown in Dulbecco’s modified Eagle’s medium (DMEM) or Lysogeny Broth (LB) at an OD_600_ of 1. Total extracts were obtained from bacterial pellets and separated by 12% native PAGE. The GrlR_2_-GrlA and GrlR-GrlR complexes and the GrlR monomer (indicated by arrows) were identified by western blotting using anti-FLAG monoclonal antibodies. **(B)** The same cell extracts were also separated by 12% SDS-PAGE and transferred to a PVDF membrane for western blotting to confirm GrlR-3xFLAG and GrlA-3xFLAG expression in the different strains using anti-FLAG antibodies.

**FIGURE 8:**
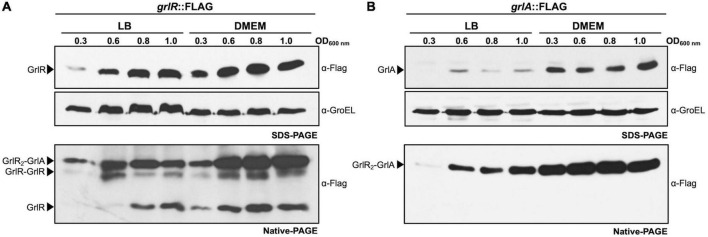
GrlR and GrlA are expressed under both repressing and inducing conditions and interact to form the GrlR_2_-GrlA heterotrimer. The enteropathogenic *Escherichia coli* (EPEC) *grlR*:3xFLAG **(A)** and *grlA*:3xFLAG **(B)** strains were grown in 50 ml of Dulbecco’s modified Eagle’s medium (DMEM) or Lysogeny Broth (LB) with shaking at 37°C. Total cell extracts were collected at OD_600_ of 0.3, 0.6, 0.8, and 1.0 and separated by 12% native PAGE. GrlR_2_-GrlA and GrlR-GrlR complexes and GrlR monomer are indicated by arrows and were identified by western blotting using anti-FLAG monoclonal antibodies. The same cell extracts were also separated by 12% SDS-PAGE to evaluate the expression of GrlR-FLAG and GrlA-FLAG by western blotting using anti-FLAG antibodies. As a control for protein loading, GroEL expression was also analyzed using anti-GroEL antibodies.

**FIGURE 9:**
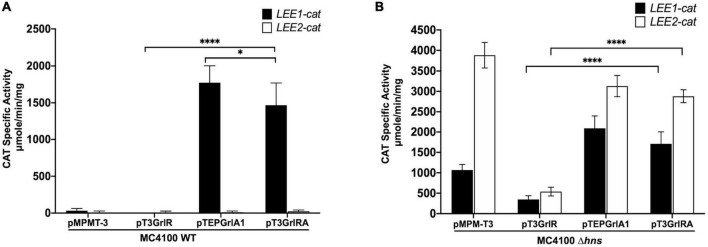
GrlR represses the expression of *LEE1* and *LEE2* operons in the absence of H-NS, GrlA and Ler. Expression of *LEE1-cat* (black bars) and *LEE2-cat* (white bars) fusions was analyzed in panel **(A)** WT *Escherichia coli* MC4100 and **(B)** MC4100 Δ*hns* (JPMC1) strains, carrying the empty vector pMPMT-3 or its derivatives pT3GrlR, pTEPGrlA1 and pT3GrlRA grown in 50 ml of Dulbecco’s modified Eagle’s medium (DMEM) with shaking at 37°C. Specific chloramphenicol acetyltransferase (CAT) activity was determined from samples collected from cultures grown in DMEM at an OD_600_ of 1. Values are an average of 3 independent experiments performed in duplicate. Error bars indicate standard deviations. Statistically different values are indicated (*p-value < 0.1; ^****^p-value < 0.0001).

**FIGURE 10:**
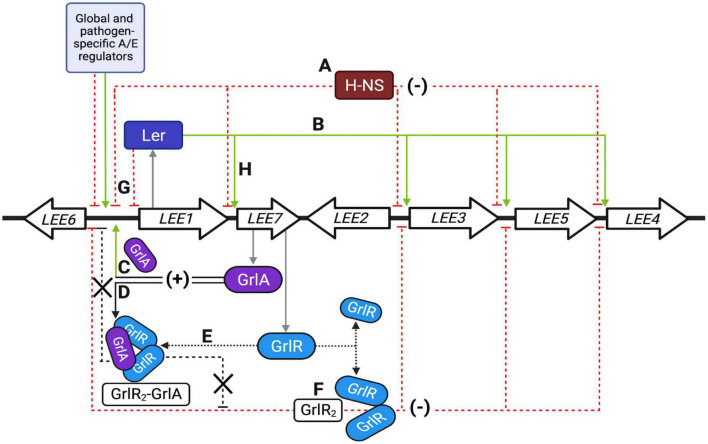
Schematic representation of the locus of enterocyte effacement (LEE) regulatory network. **(A–H)** Represent the primary regulatory circuits controlling LEE gene expression, as described in detail in the discussion. Green and red dashed lines indicate positive and negative regulatory pathways; black arrows indicate protein interactions. Thick arrows show a simplified representation of the seven operons previously reviewed in detail (Gaytan et al., 2016). Single genes are not illustrated. The first gene of the polycistronic *LEE1* operon encodes Ler, while the bicistronic *LEE7* operon codes for GrlR and GrlA. The light blue box represents several other global, ancestral and pathotype-specific regulators that modulate LEE gene expression, mainly by acting, but not exclusively, on Ler expression. Other colleagues have extensively and elegantly reviewed the regulatory elements known to modulate the regulation of the LEE (Furniss and Clements, 2018; Platenkamp and Mellies, 2018; Turner et al., 2019; Serapio-Palacios and Finlay, 2020). Created with BioRender.com.

In A/E bacteria, the LEE pathogenicity island is a distinctive example of a genetic element whose expression is controlled by a complex network of global (ancestral) and horizontally acquired regulatory proteins in response to a wide variety of environmental factors (Furniss and Clements, 2018; Platenkamp and Mellies, 2018). One of the main regulatory mechanisms involved in controlling LEE gene expression is the xenogeneic silencing exerted by H-NS (Bustamante et al., 2001). As described in different enterobacteria, H-NS specifically silences gene transcription by binding to AT-rich DNA sequences of exogenous origin to maintain cell integrity but also contributes to the acquisition of beneficial sequences (Navarre, 2016). Interestingly, within the LEE, the first gene of the *LEE1* operon encodes Ler (LEE-encoded regulator), a protein belonging to the H-NS family of nucleoid-associated proteins, which is the central positive regulator controlling the expression of *LEE* genes, and genes located outside the LEE, by counteracting the silencing effect exerted by H-NS on the LEE operons (Elliott et al., 2000; Sperandio et al., 2000; Bustamante et al., 2001; Sanchez-SanMartin et al., 2001; Haack et al., 2003; Barba et al., 2005; Bingle et al., 2014). Due to its essential role, modulation of *LEE1* operon expression is key for activating or repressing all the LEE genes and non-LEE co-regulated genes; thus, its regulation is complex and multifactorial (Furniss and Clements, 2018; Platenkamp and Mellies, 2018; Turner et al., 2019).

The LEE encodes two additional regulatory proteins: GrlA (Global regulator of LEE-activator) and GrlR (Global regulator of LEE-repressor). GrlA shares homology with a small number of predicted uncharacterized proteins in different bacterial species and with CaiF, an activator of genes involved in carnitine utilization (Deng et al., 2004; Jimenez et al., 2010). A predicted helix-turn-helix (HTH) motif is found at the N-terminus of these proteins, where most of the conservation is observed. GrlA binds to the *ler* promoter to positively regulate its expression (Huang and Syu, 2008; Jimenez et al., 2010; Bustamante et al., 2011; Padavannil et al., 2013), while Ler also induces the *grlRA* operon establishing a positive regulatory loop that enhances LEE gene expression under inducing conditions (Barba et al., 2005). Point mutations at the HTH motif affect the activation function of GrlA (Jimenez et al., 2010; Islam et al., 2011; Padavannil et al., 2013). Moreover, GrlA is present in the cell in a membrane-associated inactive state that responds to mechanical stimuli (Alsharif et al., 2015; Sirisaengtaksin et al., 2020).

In contrast, GrlR acts as a repressor by forming a dimeric structure of antiparallel beta-barrel subunits with a molecular mass of 29 kDa, which exerts its function as a negative regulator through the interaction with the HTH motif of GrlA, forming a complex with a molecular mass of 47.3 kDa that prevents the activation of the *ler* promoter (Deng et al., 2004; Lio and Syu, 2004; Iyoda and Watanabe, 2005; Jobichen et al., 2007; Jimenez et al., 2010; Padavannil et al., 2013). Orthologs of GrlR are found in species of the genera *Proteus*, *Morganella*, *Serratia*, *Klebsiella* and *Salmonella*, among others, sharing between 30 to 42% identity; however, these GrlR orthologs are hypothetical proteins with no assigned function yet. The ClpXP complex, an AAA + protease, positively controls LEE gene expression in EHEC through direct regulation of GrlR levels during the stationary phase of growth (Iyoda and Watanabe, 2005). Moreover, Hfq, an RNA chaperone, together with the small RNAs MgrR, RyhB and McaS, negatively modulate LEE gene expression in EPEC at the post-transcriptional level by destabilizing the *grlRA* mRNA and, consequently, the expression of GrlR and GrlA (Hansen and Kaper, 2009; Bhatt et al., 2017; Sudo et al., 2022). Furthermore, GrlA and GrlR also regulate the expression of genes located outside the LEE, such as the hemolysin and flagellar genes in EHEC (Iyoda et al., 2006; Saitoh et al., 2008), as well as some non-LEE encoded effector genes in EPEC (Garcia-Angulo et al., 2012), indicating that during the evolution of A/E organisms other genes were incorporated into the Ler-GrlRA regulatory network to coordinate other functions that enhanced the pathogenic capabilities of these bacteria.

Despite the current knowledge, the mechanisms underlying the interplay between GrlA and GrlR in regulating LEE and non-LEE genes under different environmental conditions are still poorly understood. In this work, we show that in addition to the notion that GrlR functions as a repressor by forming a complex with GrlA, it can also act as a repressor of LEE genes independently of this interaction, while GrlA has a dual role as a positive regulator by also antagonizing GrlR through protein-protein interactions. Our data further illustrate that LEE gene expression is negatively regulated at two levels mediated by global (H-NS) and EPEC-specific (GrlR) regulators.

## Materials and methods

### Bacterial strains, plasmids, and culture conditions

The bacterial strains and plasmids used in this study are listed in Supplementary Table 1. Bacteria were routinely cultured in Lysogeny Broth (LB) or in Dulbecco’s modified Eagle’s medium (DMEM) containing glucose [0.45% (wt/vol)] and L-glutamine (584 mg/l), but not sodium pyruvate (Gibco-BRL Life Technologies, Waltham, MA, USA), supplemented with 1% LB. When necessary, antibiotics were added at the following concentrations: kanamycin (Km) 30 μg ml^–1^, ampicillin (Ap) 100 μg ml^–1^, tetracycline (Tc) 12 μg ml^–1^, streptomycin (Sm) 100 μg ml^–1^ and chloramphenicol (Cm) 25 μg ml^–1^. To induce the expression of the LEE virulence genes, bacteria were grown either in 50 ml DMEM under shaken or static + 5% CO_2_ conditions at 37°C, while shaken LB broth was used as the non-inducing or repressing condition (Martinez-Laguna et al., 1999; Bustamante et al., 2001, 2011).

### DNA manipulations

Standard genetic and molecular techniques were applied as described previously (Sambrook and Russell, 2001). Restriction enzymes were obtained from Thermo Scientific, Waltham, MA, USA and used according to the manufacturer’s instructions. PCR reactions were performed in 50 μl using Platinum Taq DNA polymerase (Invitrogen, Waltham, MA, USA). The oligonucleotides used for PCR amplification were synthesized at the Oligonucleotide Synthesis Facility of the Instituto de Biotecnología/UNAM, Cuernavaca, México, and are listed in Supplementary Table 2.

### Construction of plasmids

To construct the pT3GrlR plasmid, a DNA sequence including the RBS and the coding region of *grlR* was amplified by PCR using the GREPKE-F/GREPX-R oligonucleotide pair. The product obtained was digested with *Kpn*I-*Xho*I enzymes and ligated into vector pMPM-T3 (Mayer, 1995), digested with the same enzymes. To generate plasmids pT3GrlRA, pT3GrlRA/I44A, and pT3GrlRA/R54A fragments were amplified by PCR with the oligonucleotides XHINTERGRLAF/HIGRLAR (Supplementary Table 2), using plasmids pTEPGrlA1, pTEPGrlA1/I44A, and pTEPGrlA1/R54A as templates (Jimenez et al., 2010), respectively. The resulting products were digested with *Xho*I-*Hin*dIII and cloned into the pT3GrlR plasmid digested with the same enzymes.

The pDnaK-CAT plasmid was constructed by PCR amplifying a fragment comprising from position −394 to + 127 of the transcription start site of the *dnaK* gene, using the DnaKF/DnaKR oligonucleotides. The product was digested with *Bam*HI-*Hin*dIII enzymes and cloned into the pKK232-8 vector (Pharmacia Biotech) (Brosius, 1984), previously digested with the same enzymes.

Chromosomal DNA from the EPEC E2348/69 strain was used as the PCR template. All plasmids generated were verified by DNA sequencing and evaluated for their ability to complement the corresponding mutants through a profile of secreted proteins or western blotting against virulence proteins.

### Construction of mutants and strains expressing FLAG-tagged proteins

The *grlR* and *grlA* mutants were produced by generating chromosomal in-frame deletions of *grlR* codons 6 to 118 or *grlA* codons 6 to 132 by the *sacB* gene-based allelic exchange method as described previously (Edwards et al., 1998). Deletion of the *grlRA* operon spans from codon 6 of *grlR* to codon 132 of *grlA*. Suicide plasmids were generated by cloning PCR-amplified fragments containing the described deletions flanked by approximately 800 to 1000 bp on each side into *Xba*I/*Sac*I-digested plasmid pRE112. The resulting suicide plasmids pRE112DgrlREP, pRE112DgrlAEP, and pRE112DgrlRAEP, respectively (Supplementary Table 1), were conjugated into strains WT EPEC E2348/69, Δ*hns* (JPEP36) and Δ*ler* to generate single and double mutants (Supplementary Table 1).

To generate the chromosomally 3xFLAG-tagged strains, a modification of the λRed recombinase system was used as described previously (Datsenko and Wanner, 2000; Uzzau et al., 2001). The PCR fragment to tag the native *grlR* gene was generated using oligonucleotides grlR-FLAGH1P1 and grlR-FLAGH2P2 and the *grlA* gene using oligonucleotides grlA-FLAGH1P1 and grlA-FLAGH2P2, and plasmid pSUB11 DNA as template. The resulting PCR products were electroporated into WT EPEC or its Δ*grlR* or Δ*grlA* mutants to generate the EPEC *grlR*:3xFLAG, *grlA*:3xFLAG, *grlR*:3xFLAG Δ*grlA* and Δ*grlR grlA*:3xFLAG (Supplementary Table 1).

Single and double mutants were verified by PCR amplification and DNA sequencing. The tagged strains contain the tag in their native gene chromosomal locations; thus, are expressed from the *grlRA* operon promoter.

### CAT assay

Chloramphenicol acetyltransferase (CAT) activity, derived from the expression of the transcriptional fusions to the *cat* reporter gene, was determined as follows (Martinez-Laguna et al., 1999). The strains containing the transcriptional fusions were grown in 5 ml of LB supplemented with antibiotics and incubated overnight at 37°C. The next day, culture pellets were adjusted to an OD_600_ = 1.0 with 1X phosphate buffered saline (PBS) (10 mM Na2HPO4, 2 mM KH2PO4, 137 mM NaCl and 2.7 mM KCl) and used to inoculate 50 ml of LB or DMEM supplemented with antibiotics with one ml of each suspension. Cultures were incubated at 37°C under shaking or static plus 5% CO_2_ growth conditions, and 1 ml samples were collected when the cultures reached an OD_600_ = 0.8 and 1.0. The cell pellet was obtained by centrifugation at 14,000 rpm for 2 minutes and washed with 1 ml of TDTT buffer (Tris-Hcl 50 mM pH 7.8 and dithiothreitol 30 μM), centrifuged again and resuspended in 0.5 ml of the same buffer. Lysis was achieved by sonication for 5 min with pulses of 10 s per minute and 5 s rest. Soluble extracts were separated from cell debris by centrifugation at 12,000 rpm for 20 min at 4°C, and 5 μl aliquots of each extract were added in duplicate to a 96-well microtiter plate followed by the addition of 200 μl of the reaction mix containing 1 mM DTNB [5,58-dithio-bis(2-nitrobenzoic acid)] (Research Organics), 0.1 mM Tris-Hcl pH 7.8, 0.1 mM acetyl-CoA (Pharmacia LKB Biotech Inc., Alameda, CA, USA), and 0.1 mM chloramphenicol (Sigma Chemical Co., Saint Louis, MO, USA). Changes in absorbance at 410 nm were recorded every 5 s for 5 min with a CERES 900C automatic microplate reader and the KC3 program set to kinetic mode. A CAT standard curve (from 0 to 2500 U/ml) was used to interpolate the activities of each sample. The protein concentration of each extract was determined by adding 10 μl of each extract in duplicate and 200 μl of the reaction mixture (25 ml of solution A + 500 μl of solution B) of the “BCA Protein Assay Kit” (Pierce) to a 96-well plate. Then incubated at 37°C for 30 min; the absorbance was read at a wavelength of 562 nm using an automatic microplate reader CERES 900 C and the KC3 program. The CAT-specific activity was determined by dividing the CAT activity by the protein concentration of each extract. The data are expressed as μmol/min/mg of protein and are the results from at least three independent assays done in duplicate. The empty vector pKK232-8 did not show measurable levels of CAT activity in any of the strains used in the study (data not shown).

### Protein secretion assay

EPEC secreted proteins were analyzed as described previously (Deng et al., 2004). Briefly, bacterial cultures in LB and DMEM were incubated at 37°C under shaking or static conditions. Triplicate 1.5 ml samples were collected per culture when the cultures reached an OD_600_ of 1.0 and were subjected to centrifugation at 17,900 x *g* for 5 min in Eppendorf tubes. Bacterial pellets were saved when convenient, and 1.3 ml of each supernatant was separated into fresh tubes and 160 μl of 100% trichloroacetic acid (TCA) was added per tube. Proteins were allowed to precipitate at 4°C overnight. Subsequently, the proteins were concentrated by centrifugation at 20,000 × *g* for 30 min and resuspended in 10 μl of 1× Laemmli loading buffer and the triplicate samples were mixed in the same tube, boiled for 5 min and resolved by 12% SDS-polyacrylamide gel electrophoresis (PAGE).

### Western blotting

Bacteria were grown in DMEM or LB under shaking or static conditions until they reached an OD_600_ of 1. Total extracts were obtained from 3 ml samples of the bacterial cultures. Cells were resuspended in 500 μl of urea solution (8 M) and lysed by sonication. Aliquots of total extracts were mixed with Laemmli buffer, boiled for 5 min and analyzed by SDS-PAGE (12% acrylamide) and, subsequently, transferred to 0.45-μm-pores-size PVDF membrane (Millipore) using a semi-dry transfer chamber (BioRad, Hercules, CA, USA).

The membrane was blocked in 10% non-fat milk and incubated with one of the following primary antibodies: a 1:10,000 dilution of monoclonal anti-Tir, a 1:7,500 dilution of polyclonal anti-intimin (kindly provided by J.A. Giron), a 1:20,000 dilution of polyclonal anti-EspA (kindly provided by J. Kaper), a 1:10,000 dilution of polyclonal anti-EscJ (kindly provided by Dr. Bertha González-Pedrajo), a 1:5,000 dilution of monoclonal anti-FLAG M2 (Sigma-Aldrich, Saint Louis, MO, USA), a 1:15,000 dilution of polyclonal anti-maltose binding protein (MBP) (New England Biolabs, Ipswich, MA, USA), a 1:10,000 dilution of polyclonal anti-DnaK (Invitrogen, Waltham, MA, USA) and a 1:50,000 dilution of monoclonal anti-GroEL (Sigma-Aldrich, Saint Louis, MO, USA). The membrane was washed with 1x phosphate buffer saline (PBS) containing 0.05% Tween 20 and incubated with a 1:10,000 dilution of secondary anti-rabbit or anti-mouse antibodies coupled to horseradish peroxidase (Thermo Scientific, Waltham, MA, USA). The membrane was developed with the commercial kit “Western Lightning Chemiluminescence Reagent Plus” (Perkin Elmer, Waltham, MA, USA) according to the manufacturer’s instructions.

### Native gel electrophoresis

EPEC E2348/69 *grlR*:3xFLAG, *grlA*:3xFLAG, Δ*grlR grlA*:3xFLAG, and *grlR*:3xFLAG Δ*grlA* strains were transformed with pT3GrlA1/I44A or pT3GrlA1/R54A and grown overnight in 5 ml of LB medium supplemented with antibiotics. The next day, 0.5 ml of these cultures were inoculated into 20 ml DMEM or LB medium supplemented with antibiotics and allowed to grow at 37°C with shaking. When the cultures reached an OD_600_ of 1.0, 5 ml samples were taken and the cells were concentrated by centrifugation at 18,000 x*g* at 4°C, resuspended in 500 μl PBS and lysed by sonication. Samples were taken at an OD_600_ of 0.3, 0.6, 0.8, and 1.0 from 50 ml cultures when needed.

Total extracts were resuspended in a non-denaturing buffer (50% glycerol, 25 mM Tris-Hcl pH 6.8 and 0.05% bromophenol Blue) and separated by 12% native PAGE at 4°C. The gel was transferred to a 0.45-μm-pore-size PVDF membrane (Millipore) and processed for western blotting.

Simultaneously, aliquots obtained from the same assays were processed for 12% SDS-PAGE and western blotting.

### Statistical analysis

Data analyses were performed by two-way ANOVA followed by Tukey’s multiple comparisons posttest using GraphPad Prism version 8.4.3 (471) for Mac OS. *P*-values < 0.05 were considered statistically significant.

## Results

### GrlR negatively regulates LEE gene expression under repression conditions

GrlR represses LEE gene expression mainly under non-permissive conditions such as growth in LB (Deng et al., 2004; Lio and Syu, 2004; Iyoda and Watanabe, 2005). To further explore this feature, we evaluated the expression of the *LEE1*, *LEE2*, *LEE4*, *LEE5*, and *dnaK* promoters fused to the *cat* reporter gene in WT EPEC E2348/69 and its Δ*grlR* non-polar deletion mutant cultured under LEE inducing (DMEM/37°C) and repressing (LB/37°C) growth conditions. Under inducing conditions, the CAT reporter activity of all LEE fusions increased between 1.5- to 4-fold in EPEC Δ*grlR* compared to the WT strain (Figure 1A); however, under repression conditions, the fold increase was higher, between 5 and 13.5 (Figure 1A). In contrast, *dnaK*-*cat* expression, used as a LEE unrelated control, was similar in both strains and growth conditions (Figure 1A).

The analysis of the T3SS-dependent protein secretion profile of WT, Δ*grlR* and Δ*ler* EPEC strains showed that, under repressing conditions (LB), the Δ*grlR* mutant abundantly secreted proteins into the medium, while the WT strain did not secrete detectable levels of LEE-encoded proteins (Figure 1B). This phenotype reversed by complementing the mutant strain with a plasmid containing the *grlR* gene (pT3GrlR) (Figure 1B). Growth under inducing conditions (DMEM) allowed secretion of LEE-encoded proteins by the WT strain and higher secretion levels by the Δ*grlR* strain. Moreover, complementing the Δ*grlR* mutant with the pT3GrlR plasmid suppressed protein secretion, even under inducing conditions (DMEM), indicating that GrlR overexpression can override the presence of GrlA under T3S permissive conditions (Figure 1B). As expected, the Δ*ler* mutant did not secrete under both growth conditions.

These results highlight the negative regulatory effect that GrlR exerts on the *LEE* genes in agreement with previous reports (Deng et al., 2004; Lio and Syu, 2004; Iyoda and Watanabe, 2005).

### GrlR represses the expression of LEE operons in the absence of H-NS

H-NS globally represses LEE-gene expression, while Ler acts as an antagonist to overcome this repression; thus, in the absence of H-NS, the role of Ler is dispensable (Friedberg et al., 1999; Leh et al., 2017; Shin, 2017). Moreover, GrlR expression from a plasmid strongly represses the expression of the LEE genes in WT EPEC even under conditions permissive to LEE expression (Deng et al., 2004; Lio and Syu, 2004; Jobichen et al., 2007). Evidence showing that GrlR and GrlA establish protein-protein interactions led to propose that GrlR negatively regulates LEE gene expression by preventing GrlA from binding to and activating the *ler* promoter (Creasey et al., 2003; Jobichen et al., 2007; Jimenez et al., 2010; Padavannil et al., 2013).

Based on such evidence, we hypothesized that overexpression of GrlR in EPEC lacking H-NS, which expresses LEE genes constitutively even under non-permissive growth conditions in a Ler- and GrlA-independent manner, would not affect LEE gene expression. Therefore, we analyzed the transcriptional activity of the *LEE1*-, *LEE2*-, *LEE4*-, and *LEE5-cat* fusions and the expression of LEE-encoded proteins in WT and Δ*hns* EPEC strains carrying or not plasmid pT3GrlR (Supplementary Table 1). Consistent with previous reports for other A/E pathogens, overexpression of GrlR in WT EPEC repressed the transcriptional activity of all LEE fusions tested (Figure 2A), as well as the expression of Tir, intimin and EspA, as shown by western blot (Figure 2B), and of EspC (Supplementary Figure 1A), an autotransporter encoded outside the LEE and whose expression is also regulated by Ler (Elliott et al., 2000; Li et al., 2004; Vidal and Navarro-Garcia, 2008). Unexpectedly, GrlR also significantly repressed the transcriptional activity of the LEE promoters in the EPEC Δ*hns* mutant (Figures 2A, B). Under these conditions, all strains similarly expressed proteins DnaK and MBP, used as loading controls, indicating that repression did not result from an unspecific pleiotropic effect in EPEC physiology due to GrlR overexpression.

To address this concept, we evaluated the effect of GrlR expressed from a plasmid under growth conditions where GrlA is not the primary activator of Ler expression. When EPEC grows in static DMEM cultures under 5% CO_2_ atmosphere at 37°C, the EAF plasmid-encoded regulator PerC is the main activator of *LEE1* promoter expression and therefore of *ler* (Bustamante et al., 2011). Thus, we analyzed the secreted protein profile and intimin and EspA expression in WT EPEC and the Δ*hns* mutant harboring plasmid pT3GrlR or the empty vector pMPM-T3, grown in shaken and static + 5% CO_2_ DMEM. As shown, GrlR expressed from pT3GrlR repressed protein secretion (Supplementary Figure 1A) and expression of EspA and Intimin in both strains and conditions, but not that of DnaK (Supplementary Figure 1B), confirming the results shown in Figure 1B.

These data suggested that GrlR can repress LEE gene expression through an alternative pathway, probably independent of its interaction with GrlA.

### The global regulator H-NS and the EPEC specific regulator GrlR act cooperatively, but at different levels, to repress the expression of LEE genes

Ler and GrlA establish a positive regulatory feedback loop that counteracts the repression exerted by H-NS (Barba et al., 2005; Jimenez et al., 2010). According to the results described above, GrlR and H-NS act cooperatively to repress the expression of LEE genes. To further explore this observation, we evaluated the expression of the *LEE1-cat*, *LEE2-cat*, and *LEE5-cat* transcriptional fusions in EPEC WT and Δ*hns* grown under inducing and repressing conditions and also analyzed the secretion profile of these strains from samples of their culture supernatants. Under inducing conditions, the activity of the *LEE2-cat* and *LEE5-cat* fusions increased about 2-fold in the absence of either of the two repressors, whereas that of the *LEE1-cat* fusion was 4.7-fold and 3.7-fold in the Δ*grlR* and Δ*hns* mutants, respectively (Figure 3A). Under repressing conditions, the derepression observed for the *LEE1*, *LEE2*, and *LEE5* promoters operons was about 13-, 7-, and 14-fold, respectively, in the absence of GrlR; whereas in the Δ*hns* mutant it was 2-, 3-, and 5-fold, respectively, relative to the activity displayed in the WT strain (Figure 3A). In agreement with these results, while the WT strain poorly secreted LEE-encoded proteins under repressing conditions (LB), the Δ*grlR* and Δ*hns* mutants showed a clear increase in protein secretion (Figure 3B).

To delve deeper into the role of both H-NS and GrlR in the negative regulation of the LEE genes, we analyzed the transcriptional activity of the *LEE2-cat* fusion and the protein secretion profile in WT EPEC and its Δ*ler*, Δ*hns*, Δ*grlR*, Δ*grlA*, Δ*grlRA*, Δ*ler*Δ*grlR*, and Δ*grlR*Δ*hns* isogenic mutants grown under inducing and repressing conditions (Figure 4). As expected, compared to the WT strain, the Δ*ler* and Δ*grlA* strains significantly downregulated *LEE2-cat* fusion expression and protein secretion, while the Δ*grlR* mutant expressed higher levels of *LEE2-cat* activity (Figures 4A, B). Interestingly, the Δ*grlRA* double mutant displayed a phenotype similar to the Δ*grlA* single mutant, confirming the important role of GrlA in *ler* activation and, indirectly, in derepression of Ler-dependent promoters that in the absence of GrlR remained repressed by H-NS (Figures 4A, B). In the Δ*grlR*Δ*hns* double mutant, the transcriptional activity of the *LEE2-cat* fusion showed expression levels similar to those observed in the Δ*grlR* and Δ*hns* single mutants under inducing conditions, while under repressing conditions, its expression was closer to the activity observed in the Δ*grlR* strain (Figure 4A). These results coincided with the secreted protein profile since, in DMEM and LB, the Δ*grlR* and Δ*hns* single mutants and the double Δ*grlR*Δ*hns* mutant showed increased secretion in contrast to the WT strain (Figure 4B). Finally, the phenotype of the Δ*grlR*Δ*ler* mutant confirmed that Ler is essential to antagonizing the repression exerted by H-NS regardless of the growth condition (Figures 4A, B).

The inclusion of the Δ*ler*Δ*hns* double and the Δ*ler*Δ*hns*Δ*grlRA* quadruple mutant in this assay was cumbersome due to the poor growth shown by these strains. To overcome this limitation, we expressed ectopically H-NS^G113D^, a dominant negative version of H-NS that carries a mutation affecting its DNA binding capacity without altering the oligomerization domain (Ueguchi et al., 1996), to inhibit H-NS activity. WT EPEC and its mutant derivatives Δ*ler*, Δ*hns*, Δ*grlR*, Δ*grlA*, Δ*grlRA*, Δ*ler*Δ*grlR*,Δ*ler*Δ*grlA*, Δ*ler*Δ*grlRA* and Δ*escN* carrying plasmid pT6-HNS/G113D, expressing H-NS^G113D^ under the control of an arabinose inducible promoter (Bustamante et al., 2008; Jimenez et al., 2010), were grown under repressing conditions with and without arabinose. We collected samples from these cultures to analyze the secreted proteins by PAGE and the expression of Tir and EscJ in total cell extracts by western blotting. In the absence of arabinose, we did not observed T3S of EspA, EspB and EspD nor Tir and EscJ expression in the WT or any strain carrying the Δ*ler* or Δ*grlA* deletions (Figures 5A, B). In contrast, upon induction of H-NS^G113D^, de-repression of T3S was evident even in the absence of Ler and/or GrlA, while the Δ*grlR* mutant showed protein secretion and Tir and EscJ expression both without or with H-NS^G113D^ induction (Figures 5A, B). The Δ*escN* strain, a mutant defective in T3S, was used as a control that did not secrete virulence proteins even upon H-NS^G113D^ induction, except for EspC, a non-T3 secreted protein, whose gene is also repressed by H-NS and derepressed by Ler (Figure 5A). In contrast, Tir and EscJ were observed in total cell extracts of this strain when H-NS^G113D^ was induced (Figure 5B).

Taken together, these results indicate that GrlR and H-NS negatively regulate the expression of LEE operons at two independent levels that are indirectly cooperative (see Section “Discussion”).

### GrlA counteracts the repressor effect of GrlR through protein-protein interactions

We previously observed that under inducing conditions, the negative effect of GrlR is counteracted when co-expressed with GrlA in *C. rodentium* (Deng et al., 2004). A possible explanation of this phenotype is that the co-expression of both proteins prevents the titration effect that overexpressing only GrlR from a plasmid has on the native levels of GrlA. However, there is also the possibility that under these conditions, the interaction between GrlR and GrlA also plays a role in preventing the GrlR-mediated independent repression described above (Figure 2 and Supplementary Figure 1).

To evaluate this possibility, we transformed plasmids pT3GrlRA, pT3GrlR, pTEPGrlA1 and the empty vector pMPM-T3 (Supplementary Table 1) into WT EPEC and grew the resulting strains under inducing and repressing conditions to analyze the secretion profile. As shown in Figure 2, GrlR overexpression repressed the LEE genes and inhibited T3S even under inducing conditions, while overexpression of GrlA overcame the repressing effect of growth in LB (Figure 6A). In turn, as observed in *C. rodentium* (Deng et al., 2004), GrlR-GrlA co-expression prevented the negative effect of GrlR, even under repressing conditions, but to a lesser extent (Figure 6A).

To further explore this notion, we took advantage of the phenotype of two GrlA mutants, GrlA/I44A and GrlA/R54A, that cannot activate the *LEE1* operon because they no longer bind to the *ler* regulatory region due to a single amino acid change in the HTH domain. Additionally, the GrlA/I44A mutant cannot interact with GrlR (Jimenez et al., 2010; Padavannil et al., 2013). The genes encoding these mutants were cloned into pT3GrlR to co-express them with WT *grlR*. WT EPEC containing the resulting plasmids pT3GrlRA/I44A or pT3GrlRA/R54A were then grown under inducing and repressing conditions to examine their T3S profile. Under inducing conditions, T3S in the presence of pT3GrlRA/I44A was similar to the secretion profile seen with pT3GrlR, as in both cases protein secretion was significantly reduced (Figure 6A). In contrast, in the presence of GrlR-GrlA/R54A, T3S was unaltered (Figure 6A), suggesting that GrlA/R54A probably prevented GrlR-mediated repression by interacting with it and not because it was over inducing Ler expression. Moreover, in LB medium, where the WT strain does not secrete T3S substrates, we observed a clear de-repression of virulence protein secretion in the presence of GrlR-GrlA/R54A, but not with GrlR-GrlA/I44A, suggesting that GrlA/R54A titrated away both the endogenous GrlR expressed from the chromosomal gene and that expressed from the plasmid (Figure 6A). To confirm the above observations, we performed a similar experiment with the EPEC Δ*grlR* strain but grown under repressing conditions (LB medium). Co-expression of GrlR with GrlA/I44A did not prevent GrlR from complementing the repression phenotype and the effect was similar to that observed for the strain with pT3GrlR (Figure 6B); however, this was not the case when GrlR was co-expressed with GrlA/R54A, as protein secretion was still observed (Figure 6B). Western blotting to detect intimin, Tir and EspA in total cell extracts of the same strains, showed similar results (Figure 6C).

These results are consistent with the notion that GrlA, in addition to its activating effect on the *ler* promoter as a DNA binding protein, could also counteract the alternative GrlR repression effect by interacting with it.

### GrlR and GrlA are expressed and interact under inducing and repressing conditions

The results described above further indicated that GrlR has a critical role as a repressor under non-permissive growth conditions for LEE expression, thus implying that EPEC expresses it in both LB and DMEM. To analyze GrlR and GrlA expression and their interaction in more detail, we grew the EPEC *grlR*:3xFLAG, *grlA*:3xFLAG, Δ*grlA-grlR*:3xFLAG and Δ*grlR-grlA*:3xFLAG strains, as well as the Δ*grlA-grlR*:3xFLAG strain carrying the plasmids encoding the GrlA/I44A and GrlA/R54A mutants (Supplementary Table 1), under inducing and repressing conditions and analyzed total cell extracts by native PAGE and western blotting from samples taken from each culture. As shown in Figure 7, the formation of the GrlR-GrlA complex occurred under both growth conditions (Figure 7A, lanes 1, 2, 7, and 8, upper band). The EPEC *grlR*:3xFLAG strain also showed the GrlR dimer and monomer (Figure 7A, lanes 1 and 7), while the banding pattern of the Δ*grlA-grlR*:3xFLAG strain, where the upper band is not present due to the absence of GrlA (Figure 7A, lines 3 and 9), confirmed the nature of the second and third bands. The EPEC *grlA*:3xFLAG strain only showed a band corresponding to the GrlR-GrlA complex (Figure 7A, lines 2 and 8), while in the absence of GrlR, GrlA-3xFLAG did not enter the gel (Figure 7A, lines 4 and 10), likely due to its isoelectric point (theoretical pI: 9.71), which changes when in complex with GrlR. Moreover, the expression of the plasmid-encoded GrlA/I44A and GrlA/R54A mutants in the Δ*grlA-grlR*:3xFLAG strain further confirmed these observations as the GrlA/I44A mutant, which does not interact with GrlR, did not form the GrlR_2_-GrlA complex (Figure 7A, lines 5 and 11) seen with the GrlA/R54A mutant (Figure 7A, lines 6 and 12). Interestingly, the GrlA/R54A mutant drove GrlR to mainly form the complex with GrlA since the dimeric and monomeric forms were not visible. In part, this may be why overexpression of GrlA in the EPEC WT strain overrode the repression in LB (Figure 6A). SDS-PAGE and anti-FLAG western blot with the same cell extracts showed the presence of the tagged proteins (Figure 7B).

Native PAGE analysis also suggested, based on the banding pattern shown by GrlR and GrlA (as discussed above), that GrlR and GrlA are expressed and interact, although not to the same extent, independently of the growth conditions. To analyze this observation in more detail, we took samples from LB and DMEM cultures of the EPEC *grlR*:3xFLAG and *grlA*:3xFLAG strains at different growth stages and analyzed them by SDS-PAGE and native PAGE followed by western blot. The qualitative analysis showed that GrlR and GrlA were expressed at moderately higher levels in DMEM than in LB; however, although enriched in DMEM, heterotrimers formed under both growth conditions independently of the expression levels of these proteins (Figures 8A, B, upper panels).

These data allowed us to hypothesize that heterotrimer formation plays a bidirectional role, on the one hand, preventing the activation of *ler* by reducing the levels of free GrlA and, on the other, antagonizing the independent repressor function of GrlR. A future more quantitative analysis of the relative concentrations of GrlA under both growth conditions, as well as the effect of these conditions on the ability of GrlA to activate the *ler* promoter and on the kinetics of GrlR-GrlA interaction, will allow a better understanding of the mechanism that regulates the expression of LEE genes in response to environmental cues.

Together, these results further supported the notion that GrlA counteracts the repressor effect of GrlR by interacting with it and showed that the GrlR_2_-GrlA heterotrimeric complex, the GrlR-GrlR dimer and the GrlR monomer are present in EPEC under both growth conditions.

### GrlR represses the expression of LEE promoters in a non-EPEC background

To further explore the notion that GrlR can repress the constitutive expression of LEE promoters independently of its interaction with GrlA, we assessed the activity of the *LEE1* and *LEE2* promoters in a background lacking not only H-NS, but also GrlA and Ler. We then transformed the empty vector pMPM-T3 or plasmids pT3GrlR, pTEPGrlA1 o pT3GrlRA into the Δ*hns* mutant of the LEE-negative lab strain *E. coli* MC4100 (JPMC1) containing the *LEE1-cat* or *LEE2-cat* fusion. The WT strain was the control, where the *LEE1-cat* fusion, but not the *LEE2-cat* fusion, was active in the presence of pTEPGrlA1 (Figure 9A), as previously shown (Bustamante et al., 2011). Interestingly, GrlR also repressed the constitutive expression of the *LEE1-cat* and *LEE2-cat* fusions in the Δ*hns* background but not when co-expressed with GrlA (Figure 9B). This result supported the proposal that GrlR also negatively regulates LEE gene expression independently of its interaction with GrlA. Also, GrlA had a dual role as a positive regulator by, in addition to its function as a *ler* activator, preventing GrlR’s negative effect, most likely by trapping it in the heterotrimer. Defining the underlying mechanism by which GrlR represses the constitutive expression of the LEE promoters will require further investigation.

## Discussion

In this study, we further investigated the role of GrlR as a repressor and the function of its interaction with GrlA in modulating LEE gene expression. Our results revealed a novel feature of its mechanism of action as GrlR also seems to act as a repressor independently of its interaction with GrlA; however, it remains to be seen if GrlR exerts this function by directly interacting with Ler-dependent promoters or with Ler-derived transcripts recognizing a common motif or indirectly by interacting with or modulating the function of a conserved element that is also present in *E. coli* K12.

A summary of the events leading to the control of LEE gene expression in response to the growth conditions is shown in Figure 10 and described below. GrlR and H-NS act independently to negatively regulate the LEE at two different levels, establishing an indirect cooperative repressive effect on the expression of LEE operons when EPEC is grown under repressing conditions (Figures 10A, F, red dotted lines). In the WT strain, H-NS silences Ler-dependent promoters (Figure 10A), while GrlR inactivates GrlA by forming GrlR_2_-GrlA heterotrimers previously shown to prevent GrlA binding to the *ler* promoter (Jimenez et al., 2010; Padavannil et al., 2013; Figure 10F). When GrlR is not present, free GrlA efficiently activates *ler* expression (Figure 10C), leading to Ler-dependent elimination of H-NS-mediated repression of LEE promoters (Figure 10B), thus resembling the phenotype shown by the double Δ*grlR*Δ*hns* mutant; however, mutants lacking *grlR* but also *ler* or *grlA*, cannot activate LEE gene expression because H-NS is still present and there is no Ler to overcome this repression.

In addition to identifying the role of GrlR as a negative regulator independent of its interaction with GrlA, our findings show that GrlA plays a dual role in the positive regulation of LEE genes by binding to the *ler* regulatory region and activating its expression (Jimenez et al., 2010; Islam et al., 2011), and antagonizing the independent repressor activity of GrlR through the formation of the GrlR_2_-GrlA heterotrimer (Figures 10C, D, respectively); thus establishing an indirect coherent-positive feedforward loop (Shoval and Alon, 2010).

How the growth conditions determine whether EPEC induces or represses the LEE genes when both proteins are present and interact needs to be clearly understood; however, current knowledge and the results described here open up interesting possibilities for future investigation. For example, GrlR, in addition to reciprocally antagonizing GrlA through their interaction, may also interact with other proteins to modulate the transcriptional activity of LEE genes or directly with DNA promoter regions or the RNA transcripts derived from Ler-regulated genes. Along with our results, reports showing that GrlA activity in EHEC is mechanoresponsive and modulated by its membrane-bound state (Sirisaengtaksin et al., 2020), that Hfq differentially regulates GrlR and GrlA synthesis (Hansen and Kaper, 2009; Bhatt et al., 2017; Sudo et al., 2022) and that ClpXP posttranslationally regulates GrlR at the stationary phase (Iyoda and Watanabe, 2005), illustrate that there is more to learn about the mechanism of action of the GrlR and GrlA duet. How the growth conditions impact the GrlR and GrlA ratio in the cell, which seems to have a profound effect on the function and interaction between GrlR and GrlA, and thus in LEE gene expression, warrants future investigation.

The presence of the GrlR_2_-GrlA complex, GrlR dimer and its monomer under both growth conditions, which is consistent with previous observations indicating that the basal activity of the *grlRA* promoter is higher than most of the LEE promoters under non-inducing conditions (Yerushalmi et al., 2014), indicate that certain levels of free GrlA are also present at any given moment during growth. Thus, the growth conditions must also play a role in determining GrlR and GrlA competency to repress or activate, respectively, when the complex is disrupted, either due to its natural dissociation or by the influence of additional environmental factors. GrlR can displace GrlA from the *ler* promoter (Padavannil et al., 2013) and here we showed that overexpression of GrlR or GrlA drives the other protein to predominantly form the GrlR_2_-GrlA complex (Figures 10D, E). In this complex, they reciprocally antagonize each other and, consequently, free GrlR or GrlA would act without competition with the other protein to either strongly repress (Figure 10F) or activate (Figure 10C) LEE gene expression, respectively. Thus, this dynamic behavior is likely modulating the activation or repression state of the LEE, whose expression has, unsurprisingly, been shown to be bimodal and render a fitness advantage to the cell (Leh et al., 2017; Ronin et al., 2017).

The acquisition of a pathogenicity island imposes on bacteria the need to adopt and adapt pre-existent and horizontally acquired regulatory mechanisms to prevent the deleterious expression of recently incorporated genes (Navarre, 2016). Although LEE gene expression is modulated by a complex assortment of global regulatory proteins, its specific activation relies on the LEE-encoded regulators Ler and GrlA. In contrast, its negative control is mediated at two levels by a global regulator (H-NS) and an EPEC-specific regulator (GrlR). In this context, self-regulation of gene expression mediated by GrlR and GrlA may have provided a fitness advantage during the acquisition of the LEE, where GrlR down-regulated its uncontrolled expression preventing detrimental effects on bacterial fitness, while GrlA counteracted this self-encoded sentinel function. It is tempting to speculate that the incorporation of pre-existing or ancestral regulatory mechanisms, such as the repression exerted by H-NS to finetune LEE gene expression further, led to the incorporation of additional regulatory proteins, such as Ler, as well as other regulators that are known to modulate Ler expression in response to various environmental, stress or metabolic signals (Furniss and Clements, 2018; Platenkamp and Mellies, 2018; Turner et al., 2019).

In line with the diversification and opposing effects of GrlR and GrlA functions, a Δ*grlR* mutant showed reduced levels of flagellin (FliC) expression and motility, while overexpression of GrlA had the opposite effect suggesting that GrlA may be acting directly as a repressor on flagellar genes (Iyoda et al., 2006; Kitagawa et al., 2011). Iyoda’s work also indicated that GrlA represses the expression of *flhD*, which encodes a master regulator of flagellar gene expression; consistently, GrlA binds to the *flhDC* regulatory region, while GrlR outcompetes this interaction (Padavannil et al., 2013), further supporting the notion that GrlA can also act as a repressor. Although details of the underlying mechanism remain undescribed, in the context of our work, the previously cited papers support the notion that the GrlR_2_:GrlA interaction plays a reciprocal modulatory role in GrlR and GrlA activities.

Overall, LEE regulation is a fine example of the integration of ancestral and horizontally acquired regulators that now comprise a complex and coordinated set of network motifs (Shoval and Alon, 2010), orchestrating a consensus response to a myriad of signals concurring to regulate the expression of the LEE genes. For example, Ler negatively regulates its own expression, establishing a checkpoint that prevents the overexpression of the LEE (Figure 10G; Berdichevsky et al., 2005), while Ler and GrlA represent a positive feedback loop (Figures 10C, H; Barba et al., 2005); however, depending on the growth conditions, activation of the *grlRA* operon by Ler may also lead to an indirect coherent-negative feedforward loop where GrlR inactivates GrlA through the formation of heterotrimers and independently represses the *ler* and other LEE promoters (Figures 10E, F; Jimenez et al., 2010; Padavannil et al., 2013). In contrast, GrlA controls an indirect coherent-positive feedforward loop by activating *ler* expression and antagonizing GrlR (Figures 10C, D; Jimenez et al., 2010; Islam et al., 2011; Padavannil et al., 2013; this work). Deciphering the unknown nuances of this intertwined regulatory network and the implications for virulence gene regulation during the transit of the pathogen from the environment to the host or within the host during the colonization process is an exciting challenge for future research.

## Data availability statement

The original contributions presented in this study are included in this article/Supplementary material, further inquiries can be directed to the corresponding authors.

## Author contributions

CL-O and JP conceived the project, designed the experiments, and wrote the original draft of the manuscript with input and edits from all authors. CL-O, AH-S, WD, and AM-L performed the experiments. CL-O, AH-S, AM-L, WD, BF, YM-L, and JP performed data analysis. BF, YM-L, and JP provided funding. All authors approved the submitted version.
