# Supplementary material for: GrlR, a negative regulator in enteropathogenic E. coli, also represses the expression of LEE virulence genes independently of its interaction with its cognate partner GrlA
Source: Front Microbiol. 2023 Feb 16;14:1063368. doi: 10.3389/fmicb.2023.1063368 (PMC9979310; doi:10.3389/fmicb.2023.1063368)
Supplement: Supplementary file 1 [file Table_1.docx]

**Table S1.** Strains and plasmids used in this study

| **Strain or plasmid** | **Genotype or description** | **Source or reference** |
| --- | --- | --- |
| EPEC strains | | |
| E2348/69 | Wild-type EPEC O127:H6, Sm^R^ | (Levine et al., 1978) |
| EPEC ∆*ler* | E2348/69 ∆*ler*::*km* | (Bustamante et al., 2011) |
| EPEC ∆*grlR* | E2348/69 carrying an in-frame deletion of *grlR* | This study |
| EPEC ∆*grlA* (JPEP35) | E2348/69 carrying an in-frame deletion of *grlA* | This study |
| EPEC ∆*grlRA* | E2348/69 carrying an in-frame deletion of *grlR and grlA* | This study |
| EPEC ∆*hns* (JPEP36) | E2348/69 ∆*hns*::*km* | Lab collection |
| EPEC ∆*grlR*∆*hns* | E2348/69 ∆*hns*::*km* derivative, ∆*hns*::*km* ∆*grlR* | This study |
| EPEC ∆*grlR*∆*ler* | E2348/69 ∆*ler*::*km* derivative, ∆*ler*::*km* ∆*grlR* | This study |
| EPEC ∆*ler*∆*grlA* | E2348/69 ∆*ler*::*km* derivative, ∆*ler*::*km* ∆*grlA* | This study |
| EPEC ∆*ler*∆*grlRA* | E2348/69 ∆*ler*::*km* derivative, ∆*ler*::*km* ∆*grlRA* | This study |
| EPEC ∆e*scN* | E2348/69 carrying an in-frame deletion of *escN* | (Gauthier et al., 2000) |
| EPEC *grlR*::*FLAG* | EPEC *grlR::3xFLAG-km* | This study |
| JPEP29 | EPEC *grlA::3xFLAG-km* | (Bustamante et al., 2011) |
| EPEC ∆*grlA* *grlR*::*FLAG* | E2348/69 ∆*grlA* derivative,  ∆*grlA* *grlR::3xFLAG-km* | This study |
| EPEC ∆*grlR* *grlA*::*FLAG* | E2348/69 ∆*grlR* derivative,  ∆*grlR* *grlA::3xFLAG-km* | This study |
| *E. coli* strains | | |
| MC4100 | F- *araD*139∆(*argF-lac*) *U169rpsL150* *relA1 flbB5301* *deoC*1 *ptsF25 rbcR,* Sm^R^ | (Casadaban, 1976) |
| JPMC1 | MC4100 ∆*hns*::*km* | (Barba et al., 2005) |
| SM10 λpir | *th*-1 *thr leu tonA lac*Y *supE*, *recA*::RP4-2-Tc::Mu KmR (*λpir*) | (Miller and Mekalanos, 1988) |
| Plasmids | | |
| pKK232-8 | pBR322 derivative containing a promoterless chloramphenicol acetyltransferase (*cat*) gen, Ap^R^ | (Brosius, 1984) |
| pler-1179 (*LEE1*) | pKK232-8 derivative carrying an EPEC *ler-cat* transcriptional fusion from nucleotides −1179 to +216 | (Bustamante et al., 2011) |
| pSepZ-11 (*LEE2*) | pKK232-8 derivative carrying an EPEC *sepZ-cat* transcriptional fusion from nucleotides -469 to +121 | (Bustamante et al., 2001) |
| pEspA-2700 (*LEE4*) | pKK232-8 derivative carrying an EPEC *espA-cat* transcriptional fusion from nucleotides -2700 to +121. | Martínez-Laguna, unpublished |
| pTIR394 (*LEE5*) | pKK232-8 derivative carrying an EPEC *tir-cat* transcriptional fusion from nucleotides -394 to +318 | (Sanchez-SanMartin et al., 2001) |
| pDnaK-cat | pKK232-8 derivative carrying an EPEC *dnaK-cat* transcriptional fusion from nucleotides -394 to +127. | This study |
| pMPM-T3 | Low-copy-number cloning vector, Tc^R^ | (Mayer, 1995) |
| pT3GrlR | pMPM-T3 derivative containing the structural *grlR* gene including the ribosomal binding site (RBS) expressed under the *lac* promoter | This study |
| pTEPGrlA1 | pMPM-T3 derivative expressing GrlA | (Jimenez et al., 2010) |
| pTEPGrlA1/I44A | pTEPGrlA1 derivative expressing GrlA I44A. | (Jimenez et al., 2010) |
| pTEPGrlA1/R54A | pTEPGrlA1 derivative expressing GrlA R54A. | (Jimenez et al., 2010) |
| pT3GrlRA | pT3GrlR derivative expressing GrlR and GrlA | This study |
| pT3GrlRA/I44A | pT3GrlR derivative expressing GrlR and GrlA I44A | This study |
| pT3GrlRA/R54A | pT3GrlR derivative expressing GrlR and GrlA R54A | This study |
| pKD46 | Plasmid containing the lambda Red system under the control of the *araB* promoter; ApR | (Datsenko and Wanner, 2000) |
| pSUB11 | Template plasmid for 3XFLAG epitope tagging | (Uzzau et al., 2001) |
| pRE112 | pGP704 suicide plasmid, pir dependent, *oriT, oriV, sacB*, Cm^R^ | (Edwards et al., 1998) |
| pRE112D*grlR*EP | pRE112 derivative containing a fragment with the in-frame *grlR* deletion from codons 6 to 118. Cm^R^ | This study |
| pRE112D*grlA*EP | pRE112 derivative containing a fragment with the *grlA* in-frame deletion from codons 5 to 132. Cm^R^ | This study |
| pRE112DgrlRAEP | pRE112 derivative containing the in-frame *grlRA* deletion from codon 6 of *grlR* to codon 132 of *grlA.* Cm^R^ | This study |
| pT6-HNS/G113D | pMPM-T6Ω derivative expressing H-NS^G113D^ | (Bustamante et al., 2008) |

References

Barba, J., Bustamante, V.H., Flores-Valdez, M.A., Deng, W., Finlay, B.B., and Puente, J.L. (2005). A positive regulatory loop controls expression of the locus of enterocyte effacement-encoded regulators Ler and GrlA. J Bacteriol 187(23), 7918-7930. doi: 10.1128/JB.187.23.7918-7930.2005.

Brosius, J. (1984). Plasmid vectors for the selection of promoters. Gene 27(2), 151-160.

Bustamante, V.H., Martinez, L.C., Santana, F.J., Knodler, L.A., Steele-Mortimer, O., and Puente, J.L. (2008). HilD-mediated transcriptional cross-talk between SPI-1 and SPI-2. Proc Natl Acad Sci U S A 105(38), 14591-14596. doi: 10.1073/pnas.0801205105.

Bustamante, V.H., Santana, F.J., Calva, E., and Puente, J.L. (2001). Transcriptional regulation of type III secretion genes in enteropathogenic Escherichia coli: Ler antagonizes H-NS-dependent repression. Mol Microbiol 39(3), 664-678.

Bustamante, V.H., Villalba, M.I., Garcia-Angulo, V.A., Vazquez, A., Martinez, L.C., Jimenez, R., et al. (2011). PerC and GrlA independently regulate Ler expression in enteropathogenic Escherichia coli. Mol Microbiol 82(2), 398-415. doi: 10.1111/j.1365-2958.2011.07819.x.

Casadaban, M.J. (1976). Transposition and fusion of the lac genes to selected promoters in Escherichia coli using bacteriophage lambda and Mu. J Mol Biol 104(3), 541-555.

Datsenko, K.A., and Wanner, B.L. (2000). One-step inactivation of chromosomal genes in Escherichia coli K-12 using PCR products. Proc Natl Acad Sci U S A 97(12), 6640-6645.

Edwards, R.A., Keller, L.H., and Schifferli, D.M. (1998). Improved allelic exchange vectors and their use to analyze 987P fimbria gene expression. Gene 207(2), 149-157. doi: S0378-1119(97)00619-7 [pii].

Gauthier, A., de Grado, M., and Finlay, B.B. (2000). Mechanical fractionation reveals structural requirements for enteropathogenic Escherichia coli Tir insertion into host membranes. Infect Immun 68(7), 4344-4348.

Jimenez, R., Cruz-Migoni, S.B., Huerta-Saquero, A., Bustamante, V.H., and Puente, J.L. (2010). Molecular characterization of GrlA, a specific positive regulator of ler expression in enteropathogenic Escherichia coli. J Bacteriol 192(18), 4627-4642. doi: 10.1128/JB.00307-10.

Levine, M.M., Bergquist, E.J., Nalin, D.R., Waterman, D.H., Hornick, R.B., Young, C.R., et al. (1978). Escherichia coli strains that cause diarrhoea but do not produce heat-labile or heat-stable enterotoxins and are non-invasive. Lancet 1(8074), 1119-1122.

Mayer, M.P. (1995). A new set of useful cloning and expression vectors derived from pBlueScript. Gene 163(1), 41-46.

Miller, V.L., and Mekalanos, J.J. (1988). A novel suicide vector and its use in construction of insertion mutations: osmoregulation of outer membrane proteins and virulence determinants in Vibrio cholerae requires toxR. J Bacteriol 170(6), 2575-2583. doi: 10.1128/jb.170.6.2575-2583.1988.

Sanchez-SanMartin, C., Bustamante, V.H., Calva, E., and Puente, J.L. (2001). Transcriptional regulation of the orf19 gene and the tir-cesT-eae operon of enteropathogenic Escherichia coli. J Bacteriol 183(9), 2823-2833.

Uzzau, S., Figueroa-Bossi, N., Rubino, S., and Bossi, L. (2001). Epitope tagging of chromosomal genes in Salmonella. Proc Natl Acad Sci U S A 98(26), 15264-15269.
