# Supplementary material for: GrlR, a negative regulator in enteropathogenic E. coli, also represses the expression of LEE virulence genes independently of its interaction with its cognate partner GrlA
Source: Front Microbiol. 2023 Feb 16;14:1063368. doi: 10.3389/fmicb.2023.1063368 (PMC9979310; doi:10.3389/fmicb.2023.1063368)
Supplement: Supplementary file 2 [file Table_2.docx]

**Table S2.** Primers used in this study

| **Primer** | **Sequence (5´- 3´)^a, b^** |
| --- | --- |
| **GREPKE-F** | CGGGGTACCAAGAAGGAGAATTCATGATTATGAAGGATGGCATCTAT (KpnI-EcoRI) |
| **GREPX-R** | ATAAATAACTCGAGATAAAAAACATAC (XhoI) |
| **XHINTERGRLAF** | CCCGGCTCGAGTTATTTATCAAATAAAAAG (XhoI) |
| **HIGRLAR** | ACCCGGGAAGCTTCGTCTAACTCTCCTT (HindIII) |
| **DnaKF** | CCGCGGATCCAAGGATTCTCTTAGTGGGA (BamHI) |
| **DnaKR** | TATAGGGAAGCTTGATCGCCTTCGGCGTTCTCC (HindIII) |
| **EP-DR8553** | TTTCTGGTTCTAGAAGCATGAGGGAC (XbaI) |
| **EP-DR9653** | CCCGGCTCGAGATCCTTCATAATCATAAC (XhoI) |
| **EP-DR9993** | CCCGGCTCGAGCAAGTTTATTTAGAATAA (XhoI) |
| **EP-DR11096** | ATGCCATCAGAGCTCATCAAATCATGAAG (SacI) |
| **EP-DA9195** | CCCATCTTCAGAGCTCTTAAATTTGC (SacI) |
| **EP-DA10080** | CCGGGCTCGAGATTTTTAGATTCCATTTT (XhoI) |
| **EP-DA10452** | GATCTTGCTCGAGAAAAAGGAGAGTTAG (XhoI) |
| **EP-DA11311** | GGAAATTTAGCTCTCTAGAGGATATTT (XbaI) |
| **grlR-FLAGH1P1** | TTAATTATTAAAAACACAGGAATGCCACAAGTTTATTTAGAAgactacaaagaccatgacgg |
| **grlR-FLAGH2P2** | TTTATTTTTATTCTTCTATAAAATATACTCAAAAAATTACGCcatatgaatatcctccttag |
| **grlA-FLAGH1P1** | AATATCTGGAACGAAATGATCTTGAGGCGGAAAAAGGAGAGTgactacaaagaccatgacgg |
| **grlA-FLAGH2P2** | TTTATTTTTATTCTTCTATAAAATATACTCAAAAAATTACGCcatatgaatatcctccttag |

1. Sequences in lower cases correspond to the template plasmid pSUB11.
2. Underlined sequences indicate restriction sites for the enzymes indicated in parenthesis.
