# Supplementary material for: GrlR, a negative regulator in enteropathogenic E. coli, also represses the expression of LEE virulence genes independently of its interaction with its cognate partner GrlA
Source: Front Microbiol. 2023 Feb 16;14:1063368. doi: 10.3389/fmicb.2023.1063368 (PMC9979310; doi:10.3389/fmicb.2023.1063368)
Supplement: Supplementary file 3 [file Presentation_1.PPTX]

## Slide 1
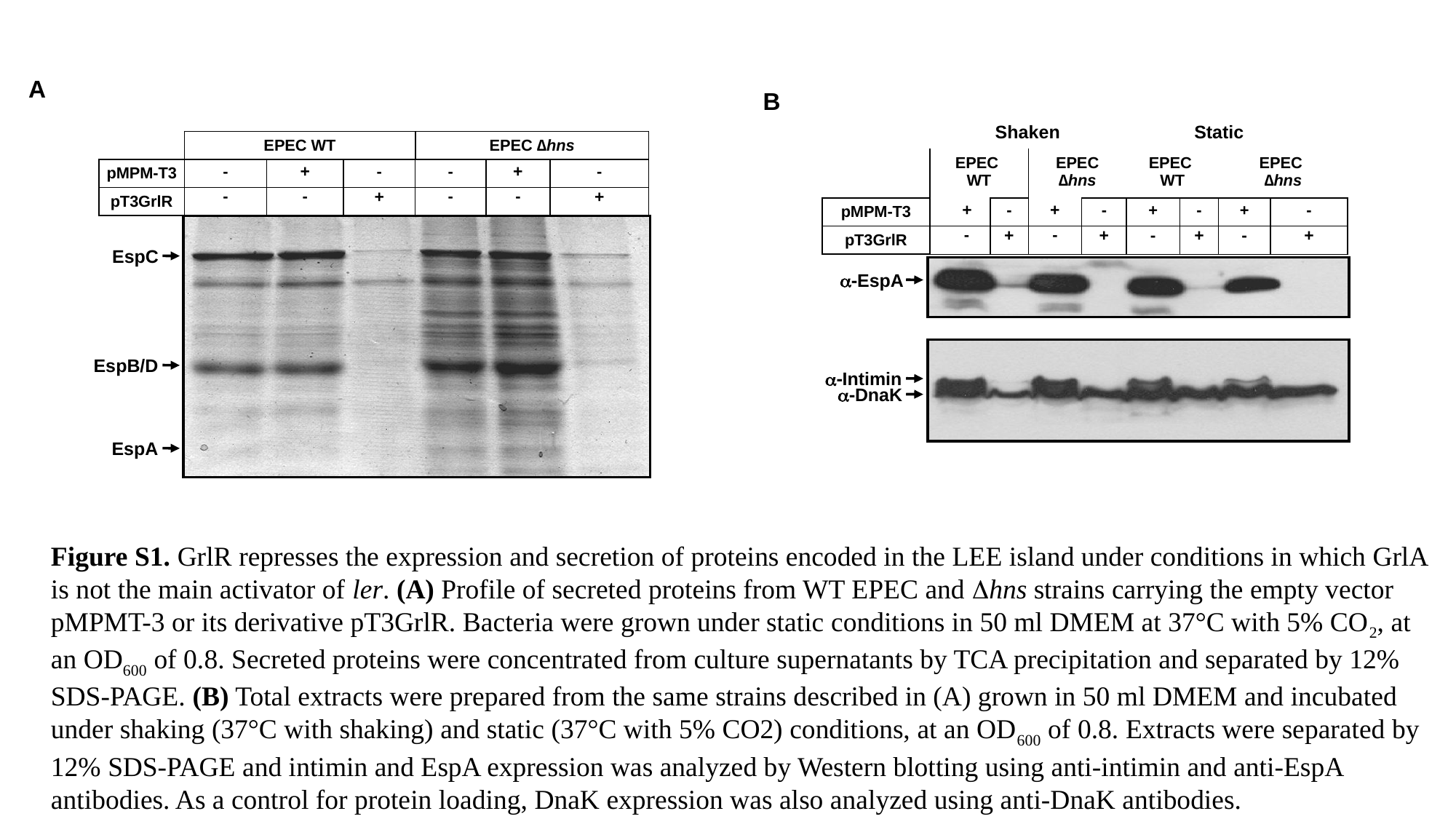

A
B
Shaken
Static
| | EPEC WT | | | EPEC ∆hns | | |
| --- | --- | --- | --- | --- | --- | --- |
| pMPM-T3 | - | + | - | - | + | - |
| pT3GrlR | - | - | + | - | - | + |
| | EPEC WT | | EPEC ∆hns | | EPEC WT | | EPEC ∆hns | |
| --- | --- | --- | --- | --- | --- | --- | --- | --- |
| pMPM-T3 | + | - | + | - | + | - | + | - |
| pT3GrlR | - | + | - | + | - | + | - | + |
EspC
a-EspA
EspB/D
a-Intimin
a-DnaK
EspA
Figure S1. GrlR represses the expression and secretion of proteins encoded in the LEE island under conditions in which GrlA is not the main activator of ler. (A) Profile of secreted proteins from WT EPEC and Δhns strains carrying the empty vector pMPMT-3 or its derivative pT3GrlR. Bacteria were grown under static conditions in 50 ml DMEM at 37°C with 5% CO2, at an OD600 of 0.8. Secreted proteins were concentrated from culture supernatants by TCA precipitation and separated by 12% SDS-PAGE. (B) Total extracts were prepared from the same strains described in (A) grown in 50 ml DMEM and incubated under shaking (37°C with shaking) and static (37°C with 5% CO2) conditions, at an OD600 of 0.8. Extracts were separated by 12% SDS-PAGE and intimin and EspA expression was analyzed by Western blotting using anti-intimin and anti-EspA antibodies. As a control for protein loading, DnaK expression was also analyzed using anti-DnaK antibodies.
